# Supplementary figures and images for: Dynamic properties of internal noise probed by modulating binocular rivalry
Source: PLoS Comput Biol. 2019 Jun 6;15(6):e1007071. doi: 10.1371/journal.pcbi.1007071 (PMC6553697; doi:10.1371/journal.pcbi.1007071)

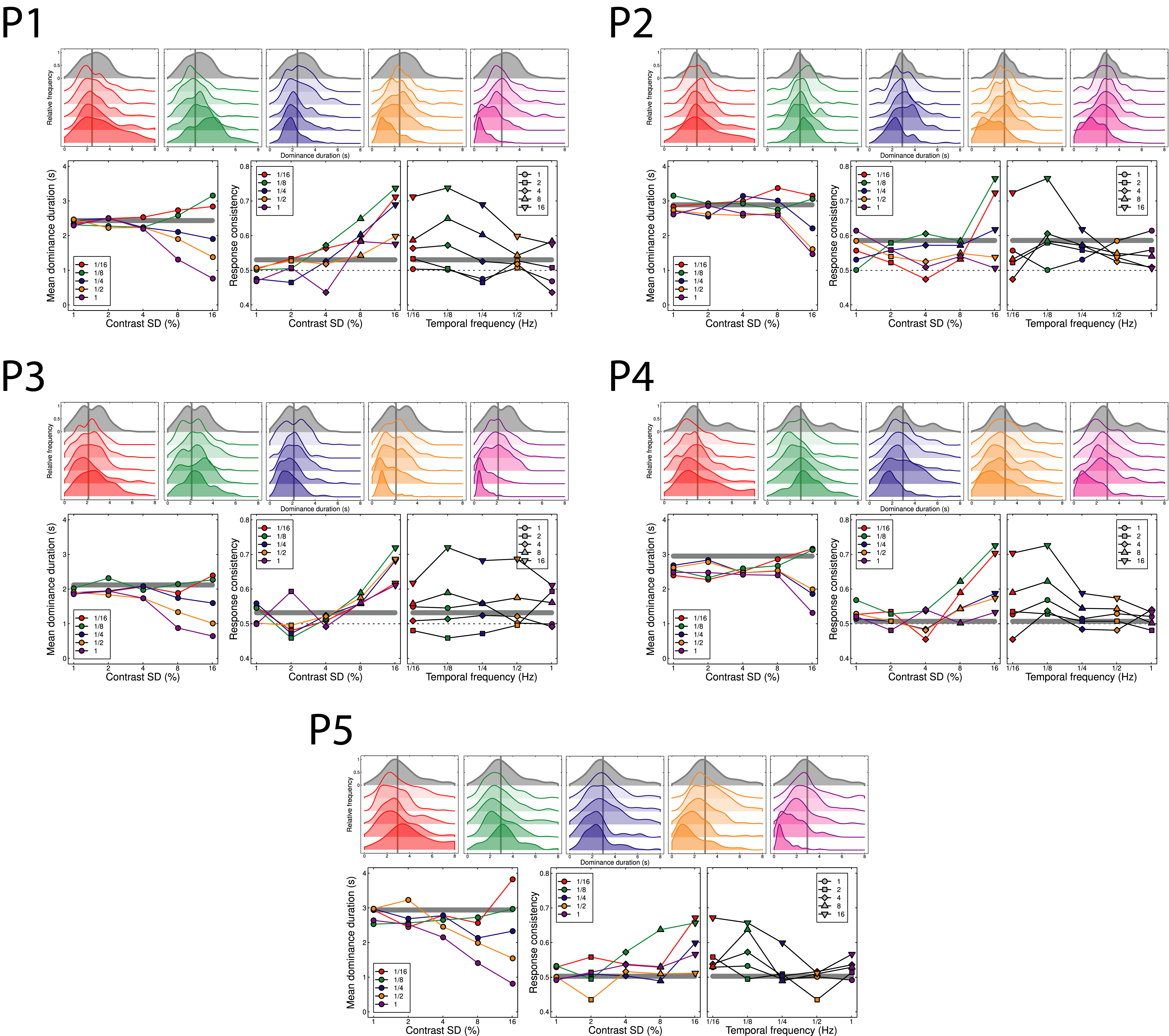

Supplement: S1 Fig — See the captions to Figs 3 and 4 for formatting details. (TIF) [file pcbi.1007071.s001.tif]

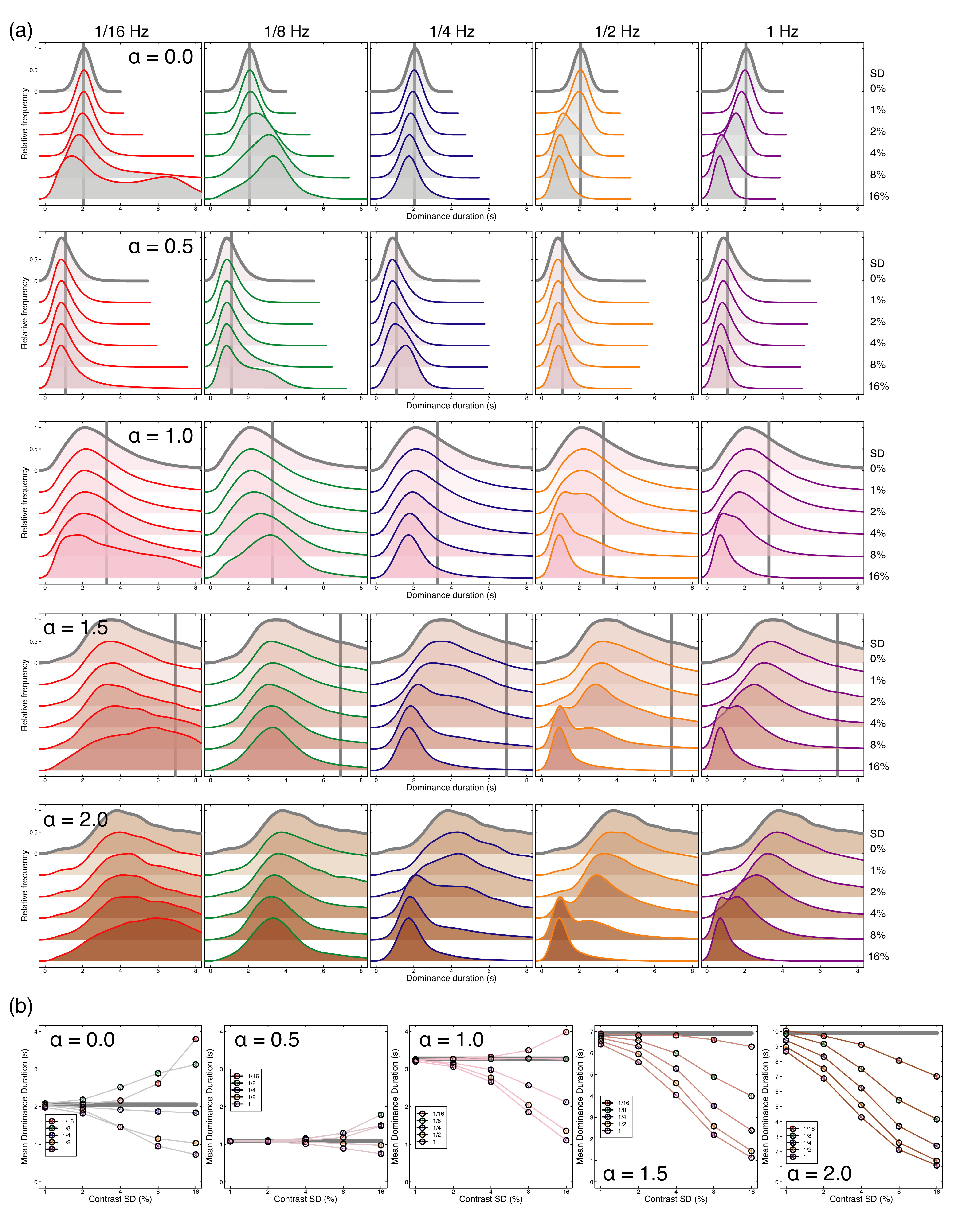

Supplement: S2 Fig — (a) Model dominance duration histograms for each of the five noise ɑs and stimulus condition as in Fig 6A. The solid line colour indicates the stimulus temporal frequency while the fill colour marks the noise ɑ. The grey vertical line marks the mean dominance duration of the 0% modulation contrast condition. For very steep slopes (ɑ = 2) the mean exceeds the x axis limit (~10s). (b) The average dominance duration for each model noise ɑ as in Fig 6B. Note the different scale for the y axis with internal noise ɑs of 1.5 and 2.0. (TIF) [file pcbi.1007071.s002.tif]

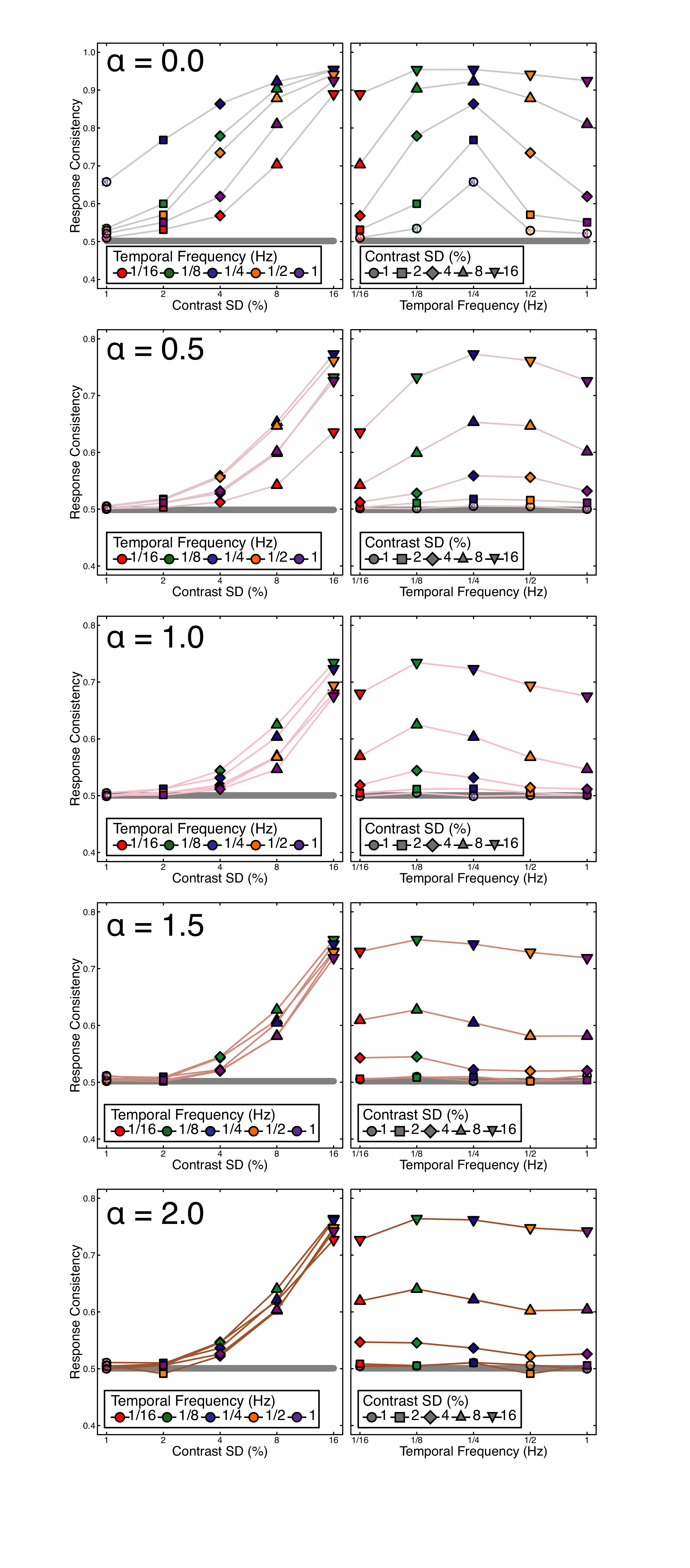

Supplement: S3 Fig — The left column charts response consistency for each modulation contrast while the right column shows the same data replotted according to modulation frequency as in Fig 6C and 6D. (TIF) [file pcbi.1007071.s003.tif]
